# Signaling by exosomal microRNAs in cancer

**DOI:** 10.1186/s13046-015-0148-3

**Published:** 2015-04-02

**Authors:** Germana Falcone, Armando Felsani, Igea D’Agnano

**Affiliations:** Institute of Cell Biology and Neurobiology, CNR, Via Ramarini 32-00015, Monterotondo, RM Italy; Genomnia srl, Via Nerviano, 31/B – 20020, Lainate, MI Italy

**Keywords:** Small non-coding RNAs, Tumor, Exosomes, Cell-to-cell communication

## Abstract

A class of small non-coding RNAs, the microRNAs (miRNAs), have recently attracted great attention in cancer research since they play a central role in regulation of gene-expression and miRNA aberrant expression is found in almost all types of human cancer. The discovery of circulating miRNAs in body fluids and the finding that they are often tumor specific and can be detected early in tumorigenesis has soon led to the evaluation of their possible use as cancer biomarkers and treatment-response predictors. The evidence that tumor cells communicate via the secretion and delivery of miRNAs packed into tumor-released microvesicles has prompted to investigate miRNA contribution as signaling molecules to the establishment and maintenance of the tumor microenvironment and the metastatic niche in cancer. In this review we highlight the recent advances on the role of exosomal miRNAs as mediators of cancer cell-to-cell communication.

## Introduction

microRNAs (miRNAs) are a family of endogenous, noncoding, tiny RNA molecules which modulate gene expression functioning both by targeting messenger RNA degradation and suppressing protein translation. Thousands of human protein-coding genes are regulated by miRNAs, indicating that miRNAs are master regulators of diverse biological systems, facilitating a rapid response of body physiology. miRNAs have been shown to regulate every aspect of cellular activity, including differentiation and development, metabolism, proliferation and apoptosis. In addition, the biogenesis and function of miRNAs are related to the molecular mechanisms of various diseases including cancer. The intensive research of the past years have demonstrated that miRNAs are not only contained into cells, but are also detectable outside them, secreted in various body fluids (e.g. serum, plasma, saliva, urine) routinely examined in patients [1,2]. It is known that extracellular miRNAs are included in small membranous vesicles or packaged with RNA-binding proteins [3]. miRNAs contained into microvesicles, and in particular into exosomes, have been shown to function as secreted signaling molecules that influence the recipient cell phenotypes. Furthermore, secreted exosomal miRNAs may reflect the molecular changes of the cells from which they originate and can therefore be seen as potential indicators for early recognition of the onset of disease. In addition, since they are relatively stable, disease-specific and can be efficiently detected in body fluids and amplified by means of molecular biology tools such as real-time PCR, they can be seen as ideal biomarkers in many pathological states including cancer.

Thanks to their unique properties and functions, miRNAs play a crucial role in human cancer development and tumorigenesis as their expression is frequently deregulated in many types of tumors, functioning either as tumor suppressors or as oncogenes (oncomiRs). Furthermore, miRNAs were shown to be responsible for cancer-related inflammation, cancer drug resistance, and regulation of cancer stem cells. Therefore, miRNAs have generated great interest as a novel strategy in cancer diagnosis and therapy [4].

## Exosomal miRNAs

The first observations that mature miRNAs are present in cell-free blood plasma and serum was made in 2008 by several independent research groups [5-8]. Later, nuclease resistant extracellular miRNAs have been found in all known biological fluids [9-12]. Since then, accumulated reports have consistently shown that extracellular miRNAs can be shielded from RNAse degradation by: (1) packaging in microvesicles (MVs) such as apoptotic bodies, shedding vesicles and exosomes; or (2) solely by complexing with AGO proteins [13-15]. Most miRNAs present in biological fluid have been found MVs-free and associated with proteins of the Ago family, which appear remarkably stable even in protease rich environment [16,17]. Some miRNA species were also found in purified fractions of high-density lipoprotein (HDL) from human plasma [18,19], although the analyzed HDL-miRNAs constituted only a minor proportion of the total circulating miRNAs. Circulating miRNAs bound by Ago proteins are apparently non-specific remnants resulting from physiological activity of the cells and cell death [15,17]. On the contrary, extracellular miRNAs included within MVs can be transferred to recipient cells, alter gene expression and mediate functional effects [20-25].

### Exosomes

Exosomes are membrane vesicles of an average 30–100 nm diameter, are formed within the multivesicular bodies (MVBs), also known as late endosomes, and released upon fusion of MVBs with the plasma membrane [26] from many different cell types in the body, such as red blood cells, platelets, lymphocytes, dendritric cells, and tumor cells. Exosome composition does not merely reflect the cells of origin, but is enriched in specific proteins, lipids and RNAs, indicating the existence of specialized sorting mechanisms, most of which are not fully understood [27]. Exosome membranes are enriched in cholesterol, sphingolipids, glycerophospholipids and ceramide [28], and bear both intraluminal and transmembrane proteins, with the same polarity as the plasma membrane. Proteins enriched in exosomes include tetraspanins (CD63, CD81) and associated proteins such as integrins, immunoglobulins and growth factor receptors; cytoskeletal proteins (tubulin, actin); ESCRT-related proteins (Alix, Tsg101); heat-shock proteins (hsp70, hsp90), and proteins involved in vesicle trafficking such as Rab GTPases, annexins and flotillin [29,30]. Exosomes are also enriched in mRNAs and small RNA species, including vaultRNA, tRNAs and miRNAs [31]. Recently, tumor-derived exosomes have been shown to carry single and double stranded DNA [32,33].

The profile of miRNAs in exosomes is specific, since particular repertoires of miRNAs are selectively sorted, while others are usually excluded. Moreover, profiling studies have revealed that exosomes of different cellular origin contain a unique expression profile of mRNAs and miRNAs, which may also differ from the signatures of their parent cells [24,34,35]. Hence, exosome content reflects the nature and even the state of the producer cell. A searchable compendium of exosomal proteins and RNA is now accessible at ExoCarta (http://www.exocarta.org/). A recent study on exosomes released from human colon carcinoma cells and purified by immunocapture with different antibodies revealed two different exosome populations distinct for both protein and miRNA composition [36]. Interestingly, miRNA passenger strands were particularly enriched in one exosomal population, suggesting that miRNA biogenesis may be interlinked with endosomal/exosomal processing [36].

The exosome composition is crucial in determining the outcome of the communication between donor and recipient cells. The mechanism of exosome-mediated cell-to-cell communication is particularly important in cancer, since tumor cells constitutively secrete exosome, which can target locally adjacent cells of the same type (autocrine effect), neighboring cells of different types (paracrine effect), or reach cells located at distant organs after entering the blood stream (endocrine effect). These exosomes play a key role in the modulation of the immune response against the tumor [37-41], the induction of angiogenesis [24,42], cell invasion and metastasis [43,44]. Tumor cells are continuously subjected to a range of stressors such as hypoxia, starvation or chemotherapeutic agents, and cancer progression depends on the ability of cells to sense and adapt to these situations. miRNA-based intercellular communication relies on critical processes. First, miRNAs must be selectively and actively secreted from cells and packaged into appropriate carriers. Second, miRNAs must be protected from circulating RNAses and transferred to targeted or receptor-specific recipient cells. Finally and most importantly, miRNAs must retain the ability to recognize and repress mRNA targets in recipient cells [45].

### miRNA sorting into exosomes

Exosome biogenesis [34,46], material cargo sorting [46,47], and release [48] involve the endosomal sorting complex required for transport (ESCRT complex), acting together with associated proteins. The ESCRT complex recognizes ubiquitinated membrane proteins and promotes their internalization into the MVBs [49]. The lipid content of exosomes appears also to play an important role in both the packaging of exosomes and their delivery to downstream tissues. The tetraspanin family of proteins, which appears specifically enriched in exosomes among extracellular vesicles, has a high affinity for both sphingolipids and cholesterol, and brings along these lipid molecules during exosome packaging [28,50]. The miRNA maturation process seems to be associated to the formation and maturation of exosomes. Whether packing of miRNAs into exosomes takes place at the pre- or mature-miRNA level has to be yet fully understood. Some authors reported that sequence motifs present in mature miRNAs are able to control their sorting into exosomes. The ubiquitous heterogeneous nuclear ribonucleoprotein A2B1 (hnRNPA2B1) recognizing these motifs specifically binds exosomal miRNAs and controls their loading into exosomes [51].

**Figure 1:**
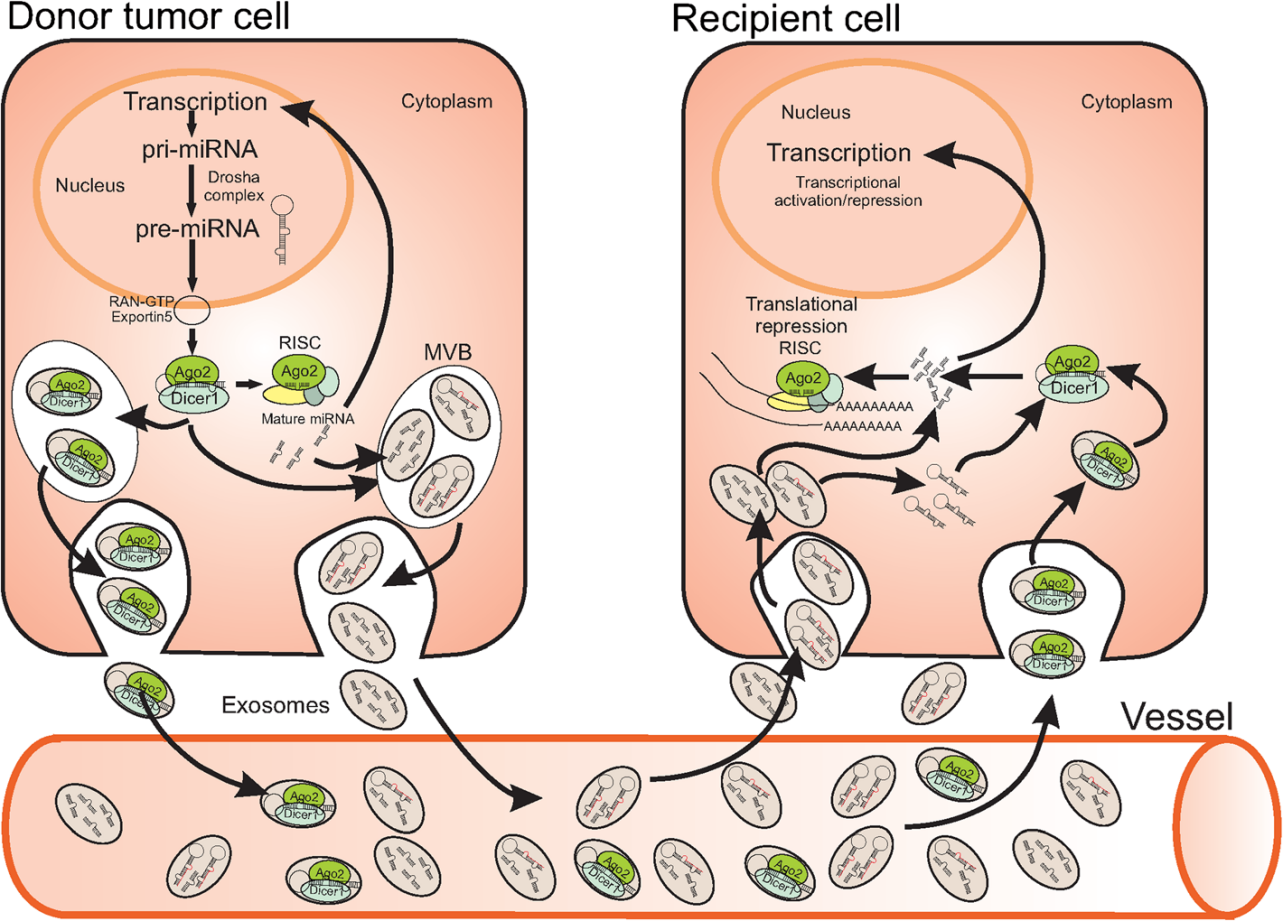
**Schematic representation of miRNA sorting into tumor cell-derived exosomes and release to recipient cells.**

It has been suggested that sorting of pre-miRNAs into exosomes could also be occurring in a sequence-dependent manner and, indeed, pre-miRNA as well as mature miRNAs are found in exosomes (Figure 1; [52-55]). A very interesting work has recently revealed that breast cancer exosomes, differently from normal cell-derived exosomes, contain pre-miRNAs along with the core RNA-induced silencing complex (RISC) proteins, namely Dicer, Ago2 and TRBP, and display cell-independent capacity to process pre-miRNAs into mature miRNAs (Figure 1; [56]). In addition to miRNAs, exosomes carry mRNAs [25], which also show a selective enrichment [24]. Exosomes content in mRNAs seems to be enriched in 3’UTR fragments [18], and this might be important for the sorting of specific mRNAs into these vesicles [57].

Recently, it has been shown that miRNA availability for exosomal secretion is controlled, at least in part, by the cellular levels of their targeted transcripts, implying that exosomal miRNA secretion is a mechanism whereby cells rapidly dispose miRNAs in excess of their targets to adjust miRNA:mRNA homeostasis [58]. While these findings suggest that miRNA sorting may be a passive mechanism, increasing data indicate that exosomes can be actively internalized by recipient cells, resulting in miRNA transfer among cells of both homo- and heterotypic nature [21,24,25,59-61].

### Exosomal miRNAs in cell-to-cell communication

The fact that exosomes are protected from the environment by their lipid bilayer makes them ideal delivery vehicles for RNAs [62]. In fact, exosomal miRNAs are more stable and resistant to degradation than cellular miRNAs [63]. In addition, exosomes can deliver multiple messages simultaneously, which make them an attractive way of exchanging specific subsets of mRNA, miRNA, or proteins between donor and recipient cells, also at a distance.

The modality of interaction of exosomes with selected target cells is still unclear. Both direct and indirect evidences exist to suggest that EVs are internalized into recipient cells, the first relying on the use of exosome fluorescent labeling, the second based on the transfer of functional miRNAs on target cells [64]. Exosomes display exquisite target cell selectivity *in vitro* and *in vivo*, which is based, at least in part, on target cell ligand interactions with exosomal tetraspanin-associated receptors. Maintenance of internalization complexes and re-use of these complexes for exosome uptake appear to be a common theme [65,66]. Importantly, the engagement of protein complexes in internalization prone membrane domains provides an explanation for the target cell selectivity that is difficult to imagine relying exclusively on single adhesion molecules, which frequently are expressed on many cells [67]. The binding of exosomes to the surface of recipient cells is mediated by the classical adhesion molecules involved in cell–cell interactions, such as integrins and ICAMs. However, other molecular pairs more specific to the exosome membrane, such as TIM-binding phosphatidylserines, carbohydrate/lectin receptors and heparan sulfateproteoglycans (HSPGs), could be involved as well [67]. To deliver their content, exosomes attached to a recipient cell can either fuse with the cell membrane, directly releasing their cargo into the cytoplasm, or get internalized by endocytic pathways. Depending in the phagocytic and endocytic capacity of the recipient cells, exosomes can be internalized by clathrin-dynamin-caveolae-dependent endocytosis, pinocytosis, or phagocytosis [67].

Accumulating evidences from the literature support the idea that exosomal miRNAs can act as regulators of gene expression in distant cells. Particularly in cancer, exosomes have multiple functions including promotion of local and systemic processes that lead to cell growth and dissemination, or impairment of the immune system response. miRNAs can act either as tumor suppressors or oncogenes (oncomiRs), depending on target genes and cancer types. Furthermore, a particular miRNA can exploit both tumor-suppressive and oncogenic functions depending on the cellular context of its target genes in different cancers [68].

**Figure 2:**
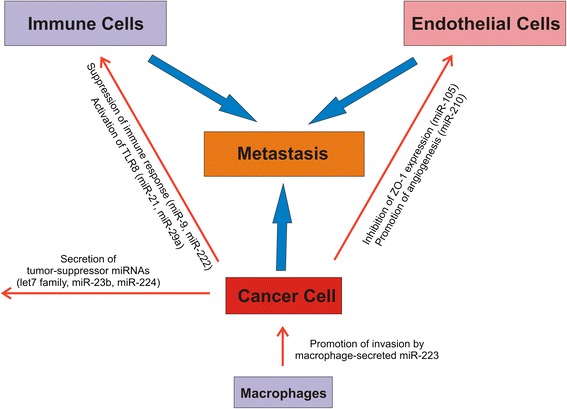
**Cancer cell-secreted exosomal miRNAs contribute to the formation of the metastatic niche.**

Table 1 and Figure 2 epitomize most significant exosomal miRNAs in cancer.

**Table 1:** **Most relevant miRNAs in cancer**

**miRNA**	**Tumor**	**Main target genes**	**Function**	**Reference**
miR-15a	Multiple Myeloma	BCL2, MYB, MYC, KRAS, CCND1, CDC25A, ERBB2, TP53, PTEN, ITGA2, VEGFA,RAB21, CADM1, DICER1	Oncogene Inhibition, Cell Cycle Control, Inhibition of Angiogenesis	[97-100]
miR-34a	Breast, Prostate, Bladder, and Brain Cancer	BCL2, MYC, MET, MEK1, E2F3, CDK4, CDK6, CD44	Cell Cycle Control, Apoptosis, Senescence, Angiogenesis	[116,117,118]
let-7 family	Breast, Colon, Gastric, Liver, Lung, Prostate, Ovarian Cancer, Chronic Myelogenous Leukemia, B Cell Lymphoma	NF2, HRAS, KRAS, NRAS, LIN28, MYC, DICER1, HMGA2, CASP3	Oncogene Inhibition, Cell Cycle Control, Cancer Stem Cell Regulation	[72,119-124,125]
miR-21	Ovarian, Lung, and Colon Carcinoma, Pancreatic and Breast Cancer	TPM1, NFIB, PDCD4, CDKN1A, FAS, TIMP3, SOX5, PTEN, BMPR2	Promotion of Cancer Cell Proliferation, Survival and Migration	[79-83,126-128]
miR-17-92 cluster	Leukemia	DICER, PTEN, E2F1, E2F2, E2F3, CDKN1A, PTEN, BCL2, CCND1, BMPR2, HOXA9	Induction of Angiogenesis, Enhance Endothelial Cell Migration	[94,129]
miR-494	Small Cell Lung Carcinoma, Breast Cancer	PTEN, CDH17, MAL	Inhibition of Apoptosis, Induction of Chemoresistance, Cell Adhesion	[89,130]
miR-210	Breast Cancer, Renal, Prostate, and Pancreatic Cancer, Lymphoma	MYC, STAT5A, HIF1A, TP53, E2F3, VEGFA, EGFR, CDKN1A, AKT1, DICER1, PTEN, CDKN1B, ERBB2, CD40, BCL2, CASP8AP2, KRAS, NOTCH1	Metastasis, Angiogenesis, Hypoxia, Tumorigenesis	[7,96,131-133]
miR-200 family	Breast Cancer	ZEB1, ZEB2, SIP1, CCND1, CDC25C, MYC, MYCN, PTEN, SIRT1, VEGFA, MET, CDKN1B, TP53, PROM1, EGFR, CD44	Tumorigenesis and Tumor Progression, Metastasis, Invasion	[92,93]
miR-9	Melanoma, Glioblastoma, Neuroblastoma	MYC, MYCN,VEGFA, MMP9, CDH1	Metastasis, Drug Resistance, Angiogenesis	[95,110,134,135]
miR-221/miR-222	Breast, Colorectal, Renal, Pancreatic, and Ovarian Cancer, Melanoma, Glioblastoma, Leukemia	KIT, PTEN, CDKN1A, CDKN1B, KRAS, DICER1, MMP1	Drug Resistance, Metastasis, Invasion, Cell Growth	[108,136-143]
miR-23b	Breast, Renal, Pancreatic, Bladder, and Prostate Cancer Thymic Lymphoma	VHL, PTEN, FAS, NISCARI, PAK2, ATG12, ZEB1, SRC, AKT	Cell Proliferation, Chemoresistance, Inhibition of Invasion and Angiogenesis	[73,75]
miR-542-3p	Pancreatic, and Cervical Carcinoma, Neuroblastoma, Colon Cancer	CDH17, TRAF4, ANGPT2, ILK, SURVIVIN	Decreased Cell Adhesion, Induction of Apoptosis, Inhibition of Proliferation and Angiogenesis	[89,144,145]
miR-125b	Breast, and Liver Cancer	LIN28B, CCND1, SOX2, MYC, CDK6,	Increase Drug Resistance, Decreased in Resistant Cells, Inhibits Cell Proliferation	[146-149]
miR-105	Breast, Ovarian, Gastric, and Prostate Cancer, Glioma, Hepatocellular Carcinoma	TJP1 DNTT, PCNA, BAX, CCND1, CDK6	Regulation of Migration, Metastasis, Proliferation, Apoptosis	[91,150-153]
miR-29	Breast, Lung, and Cervical Cancer, Neuroblastoma, B Cell Chronic and Acute Myeloid Leukemia	BACE1, DNMT3A, DNMT3B, CDC42, CDK6, TGFB3, IFNG	Modulation of Immune Response, Tumor Growth and Metastasis, Tumor Suppressor Function	[85,154-156]

## Functions of exosomal miRNAs in cancer

Exosomes have been found to act as vehicles for suppressive signals and have suppressive effects on antitumor immune responses [35,69]. For instance, miR-9 that is over-expressed in many cancers is able of inhibiting the transcription of the MHC class I gene thus preventing the recognition of tumor cells by the patient immune system [70]. Yet, miR-222 down-regulates the expression on tumor cell surfaces of intracellular cell adhesion molecule 1 (ICAM-1) whose binding to lymphocyte function-associated antigen (LFA-1) is essential for optimal activation of cytotoxic T cells, which in turn mediate tumor cell lysis [71].

In some cases, exosomal pathways might discard tumor-suppressor miRNAs that restrict metastatic progression. For example, the let-7 miRNA family was shown to be expressed at higher levels in exosomes derived from metastatic gastric cancer cells, compared to non-metastatic parental cells, suggesting that the exosomal mechanism may be used by metastatic cells to eliminate miRNAs having tumor suppressive functions, thus strengthening their aggressive behavior [72]. Another example comes from a recent paper showing that metastatic cells from bladder carcinoma secrete increased levels of miRNAs with tumor-suppressor functions (e.g. inhibition of invasion, angiogenesis, and pulmonary metastasis), including miR-23b, miR-224, and miR-921. Moreover, miRNAs, such as miR-23b, highly exocytosed from metastatic cells were reduced in lymph node metastases compared with patient-matched primary tumors [73]. These results suggest that exosome-mediated secretion of tumor-suppressor miRNAs is selected during tumor progression as a mechanism to coordinate activation of a metastatic cascade. An alternative hypothesis has been proposed to justify the relative abundance of miRNAs with predominant tumor suppressor activity in the blood of healthy individuals. According to this hypothesis, tumor-suppressor circulating miRNAs might work as a surveillance mechanism exerting continuous inhibition on tumor formation, complementing cancer immune surveillance [74]. It should be noted, however, that, in other instances, miR-23b may act as tumor promoting [75].

However, most literature reports a tumor promoting role of exosomal miRNAs, likely because tumor suppressor miRNAs are downregulated in many tumors, as well as in cancer stem cells. Many oncomiRs have been described in the past decade. For example, IL-4 activated macrophages have been shown to regulate invasiveness of breast cancer cells through exosome-mediated delivery of the miR-223 highlighting a novel communication mechanism between tumor-associated macrophages and cancer cells [76]. miR-21 has been found up-regulated in a wide range of solid tumors [77,78], and secreted in plasma exosomes from patients affected by different cancer types, such as ovarian, lung and colon carcinomas, pancreatic cancer and others, its presence being always positively correlated with tumor progression and aggressiveness [79-82]. Up-regulation of miR-21 was shown to promote cellular proliferation, survival, invasion and migration in different cancer cell lines [83], while its knock-down decreased tumor cell survival *in vitro* and tumor growth *in vivo* in a murine xenograft model, accompanied by enhanced apoptosis [84]. Interestingly, miR-21 and -29a secreted by tumor cells via exosomes have been shown to bind to toll-like receptors (TRL) on immune cells, leading to TLR-mediated NF-κB (nuclear factor kappa-light-chain-enhancer of activated B cells) activation and secretion of prometastatic inflammatory cytokines that may ultimately lead to tumor growth and metastasis [85].

It is becoming clear that tumor released exosomes contribute to both progression of primary tumors and metastases. The central role of exosomes in tumor promotion has been recently highlighted by the discovery that breast cancer exosomes can perform cell-independent miRNA biogenesis and stimulate non-tumorigenic epithelial cells to form tumors, by altering their transcriptome in a Dicer-dependent manner [56]. The metastatic process involves the manipulation of the cellular microenvironment to optimize conditions for deposition and growth both locally and at a distance for tumor colonization [86,87].

It was recently reported that melanoma exosomes can modify distant lymph nodes to facilitate melanoma growth and metastasis even in the local absence of tumor cells [88]. Exosomal miRNAs derived from metastatic adenocarcinoma cells were also involved in modulation of premetastatic organ stroma cells toward supporting tumor cell hosting. Exosomal mRNAs and miRNAs derived from tumor cells were recovered in lymph node stroma and lung fibroblasts, and were shown to significantly affect mRNA translation in the target cells, exemplified by abundant recovery of exosomal miR-494 and miR-542-3p, which targeted cadherin17 [89]. In addition to modulation of stromal cells, recent data have also demonstrated a pivotal role for cancer cell–derived exosomes in the organization of the extracellular matrix (ECM). Being rich in proteases, exosomes can modulate the ECM for degradation of collagens, laminin, and fibronectin, and this may have severe consequences on tumor and host cell adhesion, motility, and invasiveness [90].

Exosomal miRNAs can also participate in cancer metastasis by adapting the tumor niche cells. miR-105, which is characteristically expressed and secreted by metastatic breast cancer cells, is a potent regulator of migration through targeting the tight junction protein ZO-1. In endothelial monolayers, exosome-mediated transfer of cancer-secreted miR-105 efficiently destroys tight junctions and the integrity of these natural barriers against metastasis. Overexpression of miR-105 in non-metastatic cancer cells induces metastasis and vascular permeability in distant organs, whereas inhibition of miR-105 in highly metastatic tumors alleviates these effects [91]. It has been recently shown that miR-200 family members, which regulates the mesenchymal-to-epithelial transition, within extracellular vesicles secreted from highly metastatic tumor cells can be internalized by weakly metastatic cells and confer the capability of tumor growth at metastatic lesions [92,93].

The contribution of exosome in induction of angiogenesis to promote cancer metastasis is also described. For instance, it was shown that miRNA-enriched exosomes released by CD105 cancer stem cells from renal carcinomas may modify the tumor microenvironment by triggering angiogenesis and may promote formation of a pre-metastatic niche [42]. Specific exosomal miRNAs, such as those of the miR-17-92 cluster, have an important role in neoplasia-to-endothelial cell communication for regulating endothelial gene expression during tumor angiogenesis in leukemia cells [94]. It was also shown that tumor-secreted miR-9 encapsulated into microvesicles promotes endothelial cell migration and tumour angiogenesis participating in intercellular communication and function [95]. Moreover, exosomal angiogenic miR-210, known to be increased in the serum of cancer patients with malignant breast cancer, regulate the metastatic ability of cancer cells through suppression of specific target genes, which resulted in enhanced angiogenesis [96]. In addition, neutral sphyngomyelinase 2 (nSMase2) was required to regulate exosomal miRNA secretion from cancer cells and promote angiogenesis within the tumor microenvironment as well as metastasis [96]. These findings suggest that the horizontal transfer of exosomal miRNAs from cancer cells can dictate the microenviromental niche for the benefit of cancer progression. Some studies also suggest that the microenvironment may play a crucial role in regulating the pathogenesis of some tumors by modulating the expression of exosomal miRNAs. Bone marrow stromal cells modulate miR-15a, which act as putative tumor suppressor in multiple myeloma cells thus facilitating the progression of this tumor [97-100].

The exosomal miRNA profiling of sera from cancer patients versus healthy individuals has often revealed important differences in relation to tumor progression, highlighting a possible use of these miRNAs as disease prognostic biomarkers [101,102]. In addition, many tumors displaying drug resistance show alterations in the expression of miRNAs. The up- or down-regulation of miRNAs affects the expression of several target proteins (drug targets, transporters, cell cycle- and apoptosis-related components, key signaling pathway components involved in drug response), determining changes in drug sensitivity through different molecular mechanisms [103,104]. Moreover, different studies indicate that exosomes act as vehicles for exchange of genetic cargo between heterogeneous populations of tumor cells, generating a way of transmitting drug resistance [105-107]. Recently Chen and colleagues reported that exosomes from drug resistant breast cancer cells are capable of delivering a subset of miRNAs (miR-100, miR-222 and miR-30a) to sensitive cells [108]. miR-34a, detected as both intracellular and exosomal biomarker, was recently found also to influence prostate cancer cell response to docetaxel by regulating anti-apoptotic BCL-2 gene [109]. In addition, the inhibition of miR-9 expression in chemoresistant glioblastoma multiforme (GBM) cells, by transferring to GBM cells miR-9 microvesicles-encapsulated inhibitors, resulted in reversed expression of the multidrug transporter and sensitized GBM cells to the treatments [110].

## Conclusions

Intensive research effort is spent to find out new cancer diagnostic and prognostic tools. Representing a non-invasive approach, measurement of extracellular miRNAs in biological fluids might prove a valuable strategy. In addition, since dysregulation of miRNAs is fundamental to the pathogenesis of many cancers, they are attractive candidates for clinical application as therapeutic targets in both solid and hematological malignancies [111]. Down-regulation of target oncogenes by re-expression of tumor suppressor miRNAs, or re-expression of tumor suppressor genes by silencing oncomiRs is expected to impair tumor growth and metastasis [112]. Noteworthy, miR-34 mimic has become the first cancer-targeted miRNA drug (MRX34) entering Phase I clinical trials (ClinicalTrials.govidentifier: NCT01829971) in patients with advanced hepatocellular carcinoma [113]. A new class of drugs that specifically target miRNA pathways are being currently developed. They work either via replacement of tumor suppressed miRNAs with synthetic or viral vector encoded miRNA mimics, or via antisense-mediated inhibition of oncogenic miRNAs. However, off-target effects must be evaluated before such therapies can be safely applied to the clinic. Recently, a novel system for miRNA or antagomiR delivery applicable for both local and systemic administration with the use of mesenchymal stem cell microparticles was developed in *in vitro* models [110,114,115]. The use of miRNA-containing exosomes as cancer prognostic biomarkers and vehicles for miRNA-based therapy could be feasible and easily exploitable in clinical practice, with the goal of developing a personalized anticancer therapy.
